# Construction of an Affordable Lumbar Neuraxial Block Model Using 3D Printed Materials

**DOI:** 10.7759/cureus.6033

**Published:** 2019-10-30

**Authors:** Kevin T Riutort, William Clifton, Aaron Damon, Conrad Dove, Steven R Clendenen

**Affiliations:** 1 Anesthesiology, Mayo Clinic, Jacksonville, USA; 2 Neurosurgery, Mayo Clinic, Jacksonville, USA; 3 Neurosurgery, Mayo Clinic, Rochester, USA

**Keywords:** simulation, epidural, 3d printing, neuraxial, phantom, regional anesthesia, spinal blockade

## Abstract

Access to affordable 3D printing technology has resulted in increased interest in the creation of medical phantom task trainers. Recent research has validated the use of these trainers in simulation education. However, task trainers remain expensive, limiting their availability to medical training programs. We describe the construction of a low-cost task trainer using fused filament fabrication (FFF) printed spinal vertebrae placed in a synthetic gelatin matrix. Additionally, our model contains a realistic simulated ligamentum flavum, a removable silicone skin, as well as spinal fluid reservoir that provides a positive endpoint for intrathecal blocks. The total cost of this model was less than $400 USD. The time to 3D print the bony anatomic parts was approximately 26 hours. While we have not formally validated our model, initial impressions of tactile feel and realism were deemed positive by experienced anesthesia providers. Future work will focus on continued refinement of the model features and construction.

## Introduction

The validity of the use of medical task trainers in simulation education, particularly those used to teach lumbar neuraxial techniques, has been verified [1]. Unfortunately, the cost of commercial trainers remains high, thus limiting access to training institutions. However, in the past several years, the creation of phantom task trainers for use in medical education has gained ground. Improvements in 3D printing technology has made the construction process of "home-built" task trainers affordable and accessible [2]. We describe herein the process to create an affordable lumbar neuraxial block task trainer. The basic construction of which is fused filament fabrication printed spinal vertebrae placed in a synthetic ballistic gelatin matrix. Additionally, our model contains a realistic simulated ligamentum flavum, a removable silicone skin, and a spinal fluid reservoir that provides a positive end-point for intrathecal blocks. Our model may be constructed relatively quickly (3D printing time is under 60 hours) and at a low cost.

## Technical report

Model creation

**Figure 1:**
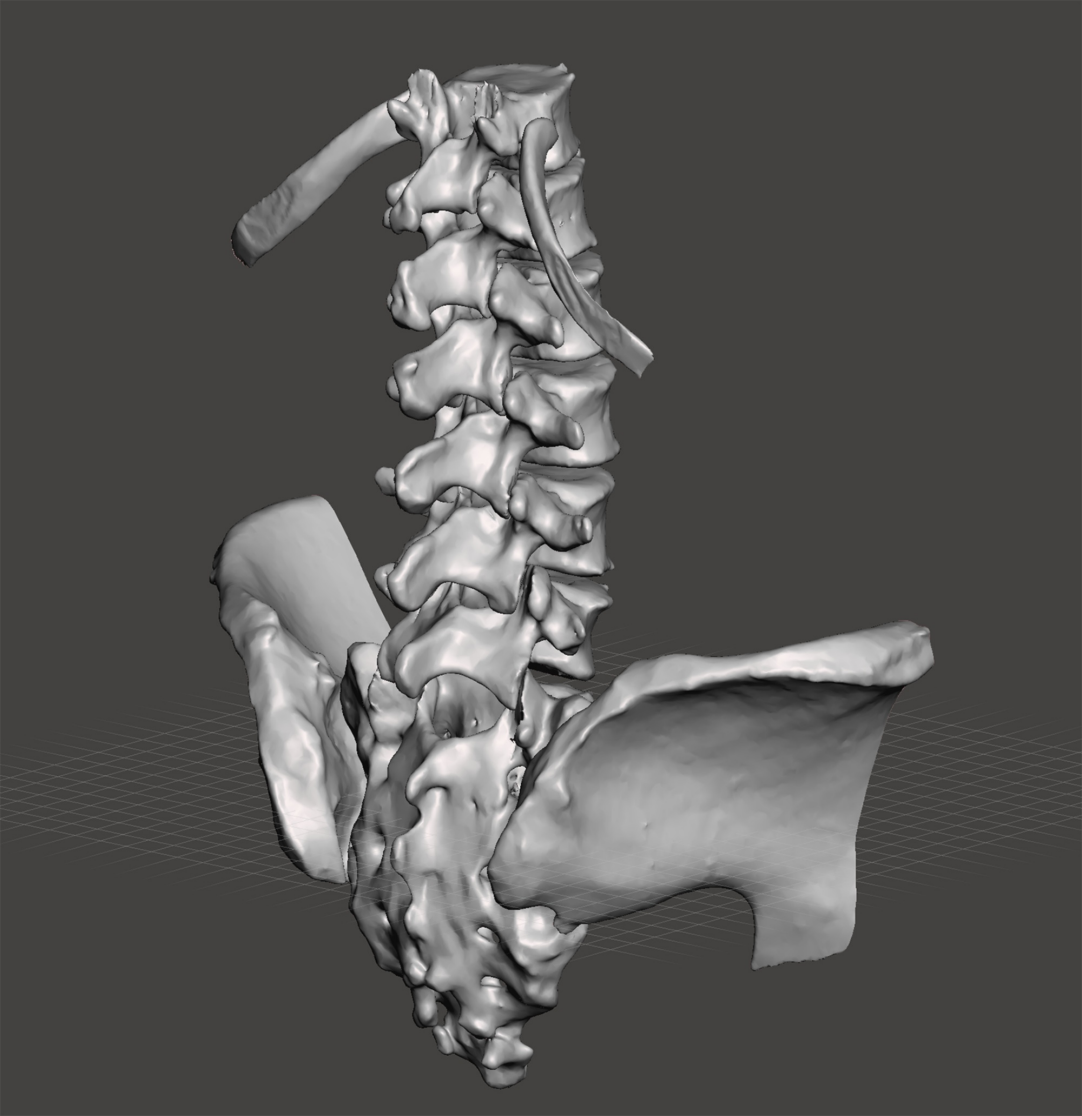
Spine model assembly created in MeshMixer.

**Figure 2:**
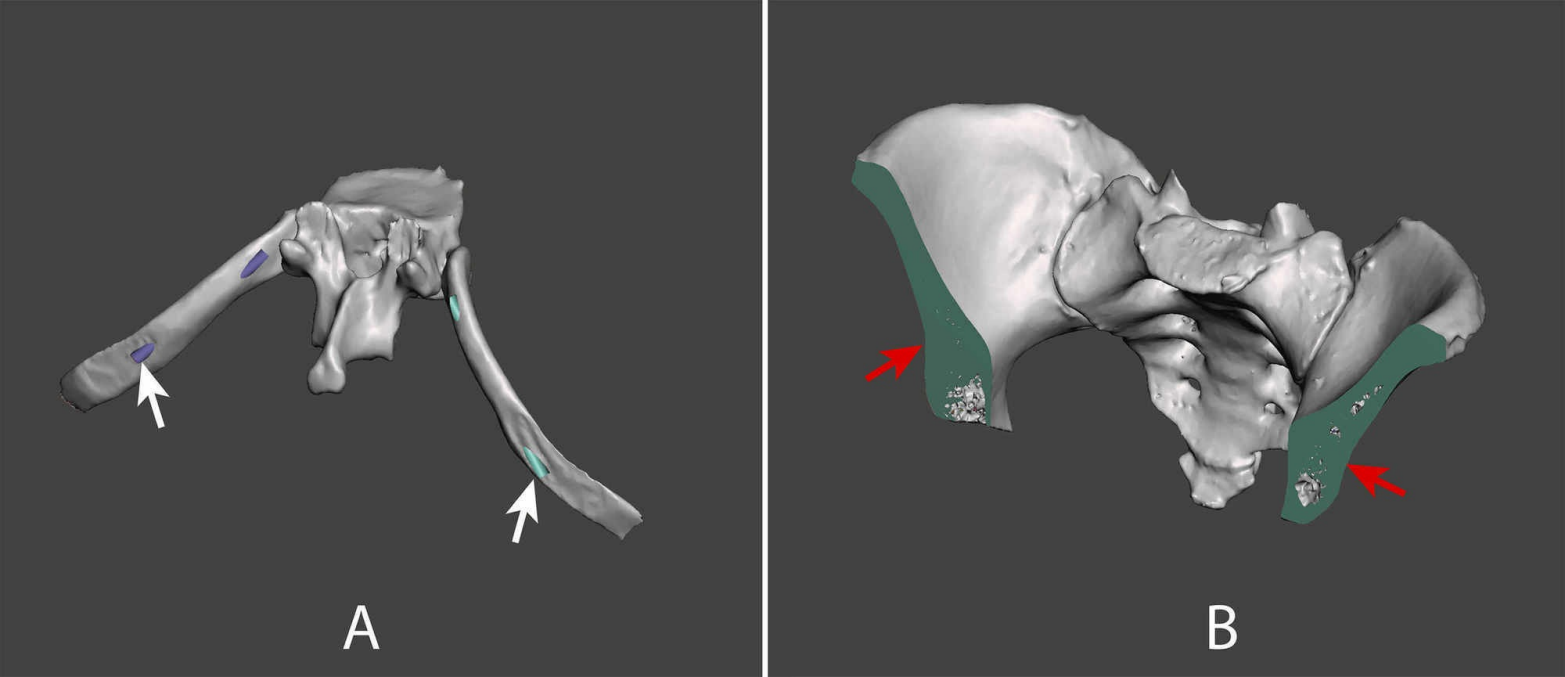
A) Vertebral level T10 model showing holes for rib armature (white arrows). B) Iliac crest model with sacrum showing cuts to reduce anterior-posterior model size (red arrows).

To create a neuraxial block task trainer, we first obtained high fidelity 3D models of relevant bony anatomy. Neuraxial blocks are often performed by anesthesiologists using only landmark techniques, thus model anatomy must accurately represent palpable surface landmarks. Using computed tomography (CT) DICOM files of lumbar anatomy, we obtained stereolithography (STL) files of the T10 through L5 vertebral bodies as well as the left and right iliac crests and the sacrum. These files were segmented using Materialise Mimics software (Materialise NV, Leuven, Belgium). We then assembled and modified the STL files using Autodesk MeshMixer (Autodesk Inc., San Rafael, CA). Figure 1 shows the MeshMixer bony lumbar anatomy assembly. Using MeshMixer, we added 3.5 mm through holes to the T10 ribs to support a 12g wire armature (Figure 2A). The T10 rib armature is necessary to prevent heat deformation when the model gelatin matrix is poured. We also modified the iliac crests models in MeshMixer by making cuts in the coronal plane, anterior to the vertebral bodies (Figure 2B). This allowed us to reduce the anterior-posterior dimension of the model.

**Figure 3:**
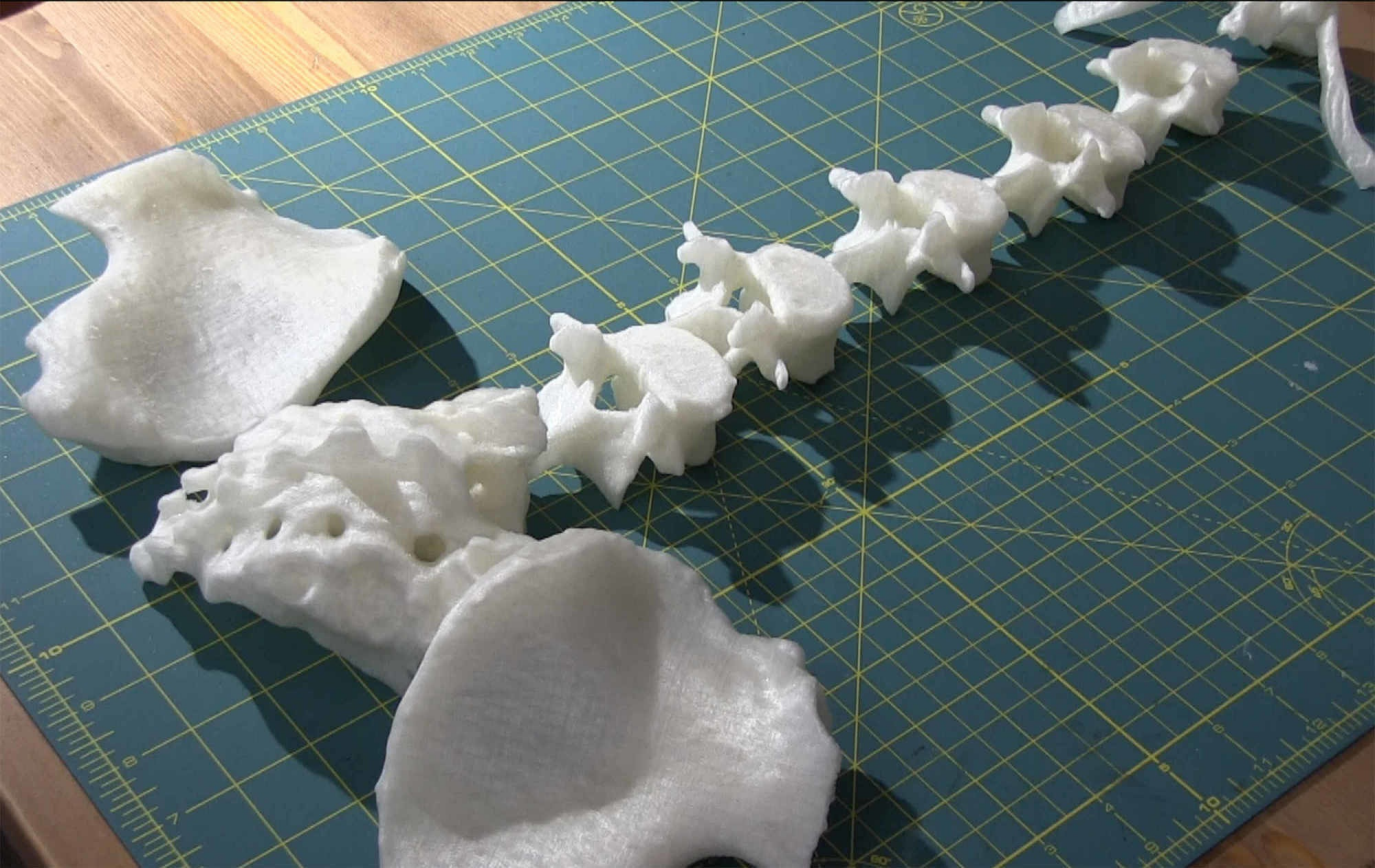
Completed 3D printed polylactic acid (PLA) parts created using a fused filament fabrication (FFF) process.

Next, we used Simplify3D (Simplify3D LLC, Blue Ash, OH) software to pre-process STL files into G-code for 3D printing. Our print process is fused filament fabrication with 0.4 mm natural polylactic acid (PLA) filament (Shenzhen Esun Industrial Co, LTD, Shenzhen, China). We printed parts with a 0.2-mm layer height, 25 percent infill, 60 C table temperature, and 205 C nozzle temperature. Our 3D printer is a Prusa i3 Mk2S printer (Prusa Research, SRO, Prague, Czech Republic) which the author built from a kit costing approximately 700 USD (this printer is also available fully assembled at a slightly higher cost). Total print time for all parts was 59.5 hours. The total cost of printing was $16.60 USD and consumed 770g of PLA filament. The completed parts are shown in Figure 3.

We then assembled the iliac crests to the sacrum using self-tapping screws. We assembled the vertebral bodies onto the sacrum using two 12g copper wire armatures. Armature holes were drilled in the parts using a hand drill. The form for the mold was constructed from a 12-inch concrete tube form (Sakrete, Inc., Atlanta, GA) with cardboard ends sealed with duct tape. However, this initial form failed as it was unable to contain the liquid ballistic gel matrix. A second form was constructed using a 0.375-inch high-density polyethylene sheet (HDPE) (King Plastic Corp., North Port, FL). The successful use of HDPE to create a mold form for ballistic gelatin has been previously described by Damon et al. [3]. The form was hand-cut with an electric jig saw and assembled with self-tapping steel screws. We then used silicone to seal any gaps in the form.

**Figure 4:**
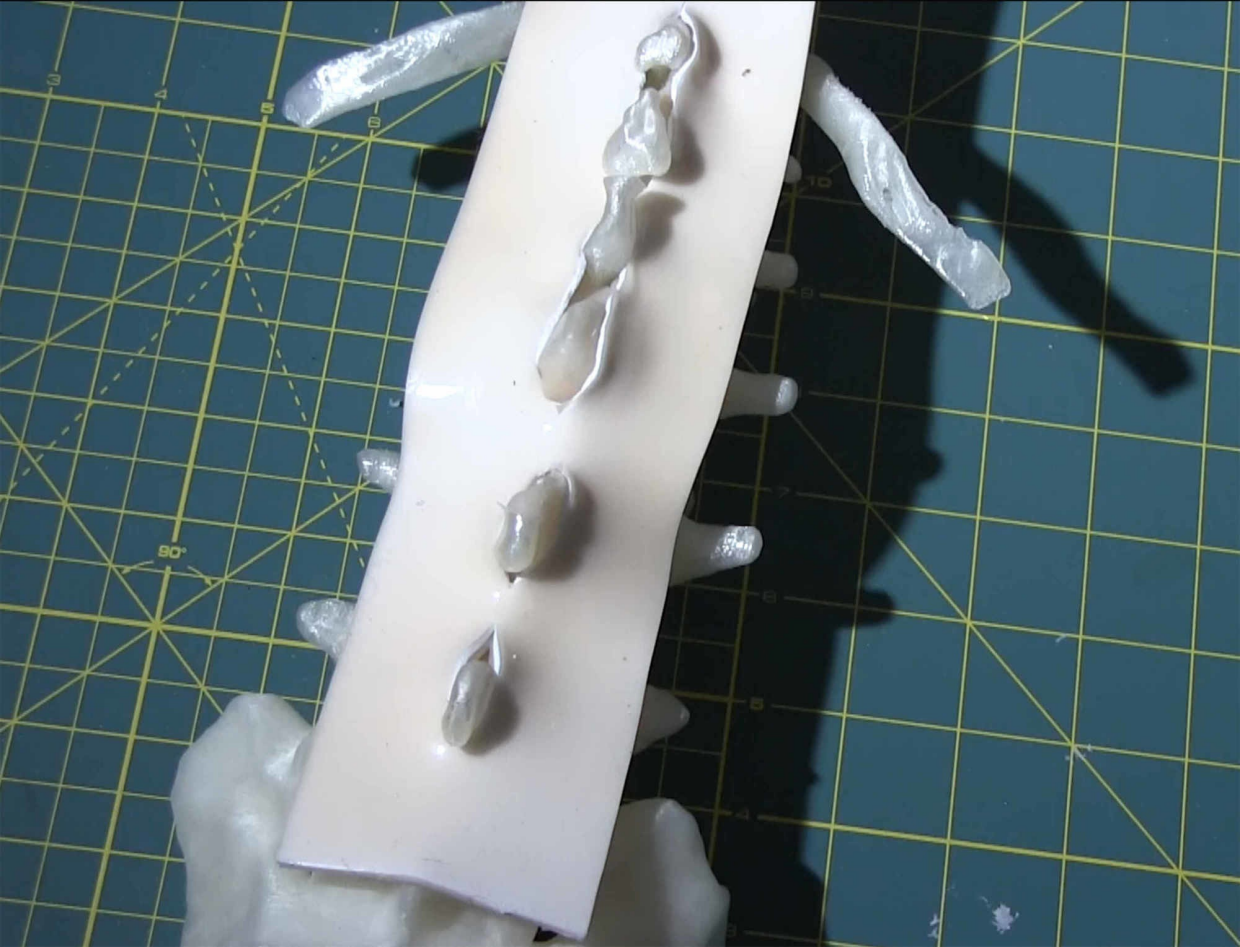
Ligamentum flavum constructed of ethylene-vinyl acetate (EVA) and silicon, installed on vertebral bodies.

The ligamentum flavum (LF) was modeled using 1.5 mm thick ethylene-vinyl acetate (EVA) foam with a silicone skin. We placed the foam in a shallow tray and poured platinum cure silicone with a shore hardness of 30 (Ecoflex™ 00-30, Smooth-On, Inc., Macungie, PA) over the foam. The final thickness of the LF was approximately 3.5 mm. Work done by Kolte et al. has shown that the mean thickness of the LF in the lumbar region (L5-S1 to L3-4), while somewhat asymmetric, varies from 3.38 ± 0.94 mm to 3.79 ± 1.24 mm [4]. Once cured (after approximately 30 minutes), we cut the LF in a 60-mm wide strip. We cut slots in the strip and placed the LF over the spinous processes in our model, trimming it to the spinal canal (Figure 4).

To hold the model in the form, we used a 12.7-mm aluminum rod placed through the spinal canal and then through each end of the form. The rod is removed after the gelatin matrix cures. This provides a space for simulated spinal fluid.

**Figure 5:**
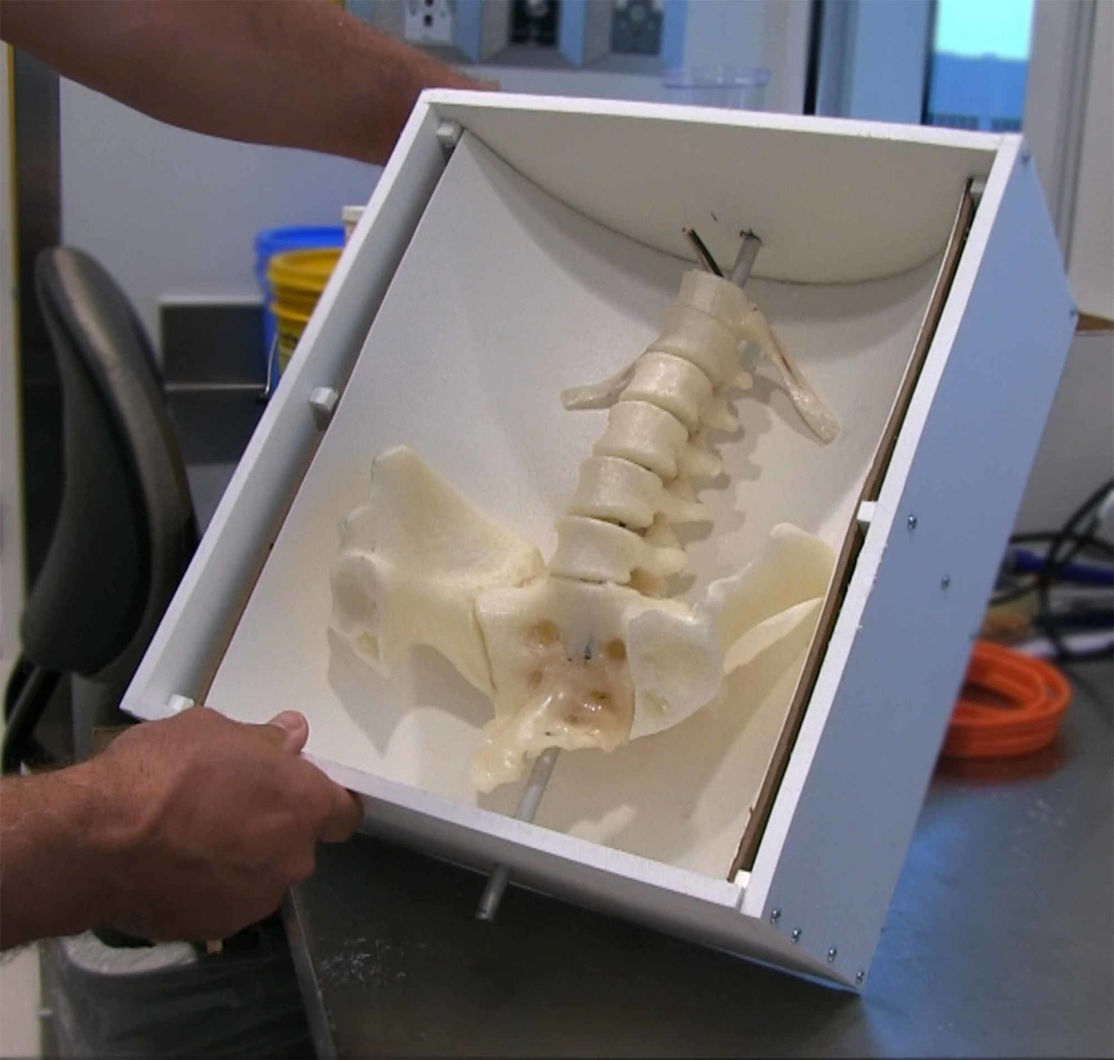
Assembled neuraxial block model ready to pour gelatin matrix.

Figure 5 shows the model ready for the gelatin pour. We chose 20% ballistic gel (Clear Ballistics, Greenville, SC). This is a reusable synthetic gel that mimics human tissue well. This product cost $33 USD per kilogram. We used 4.7 kg at a cost of about $153 USD. This ballistic gelatin needs to be heated to 121 C to 132 C for four hours prior to pouring. This is accomplished using an electric household cooking pot.

**Figure 6:**
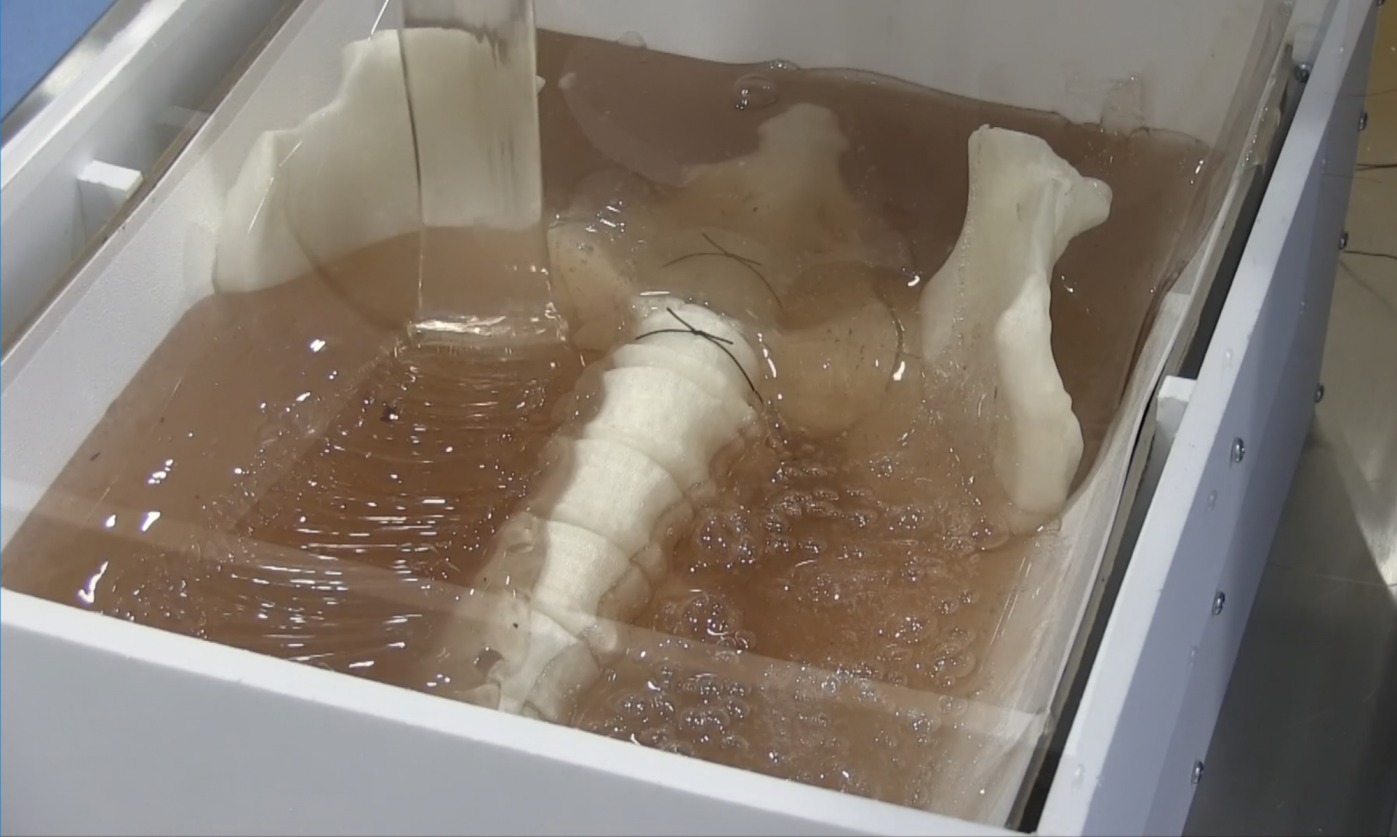
Pouring gelatin matrix.

Figure 6 shows the model during the pour. During the pour, air bubbles may become entrained in the gel matrix. Using a heat gun, we were able to remove many of these bubbles. The cure time for the gelatin is 12 hours. Once removed from the mold, we again used the heat gun to remove the air bubbles as well as smooth the gel matrix.

Finally, we created a 4-mm thick silicone skin that can be placed over the model to obscure bony anatomy.

Results

**Figure 7:**
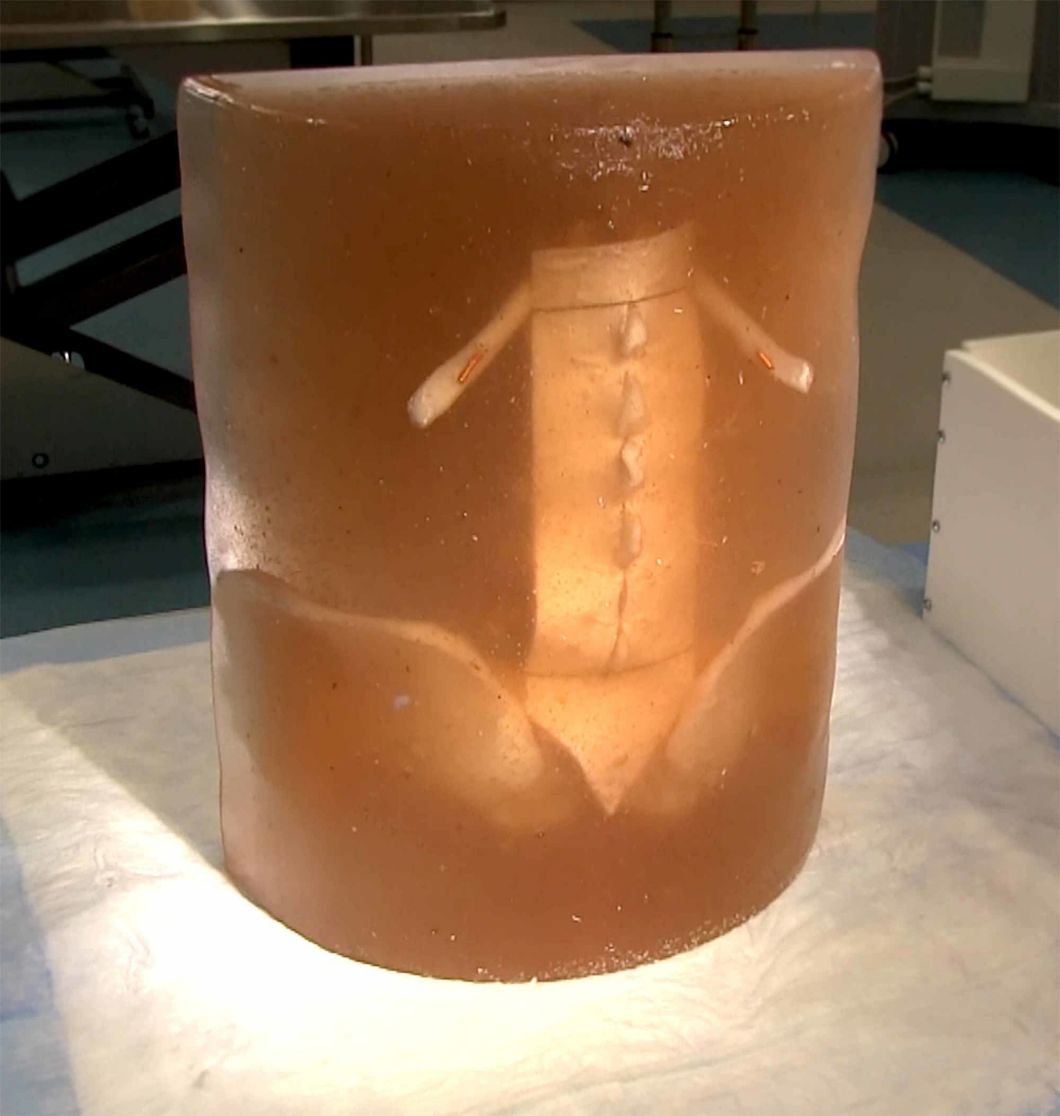
Completed lumbar neuraxial block model.

**Figure 8:**
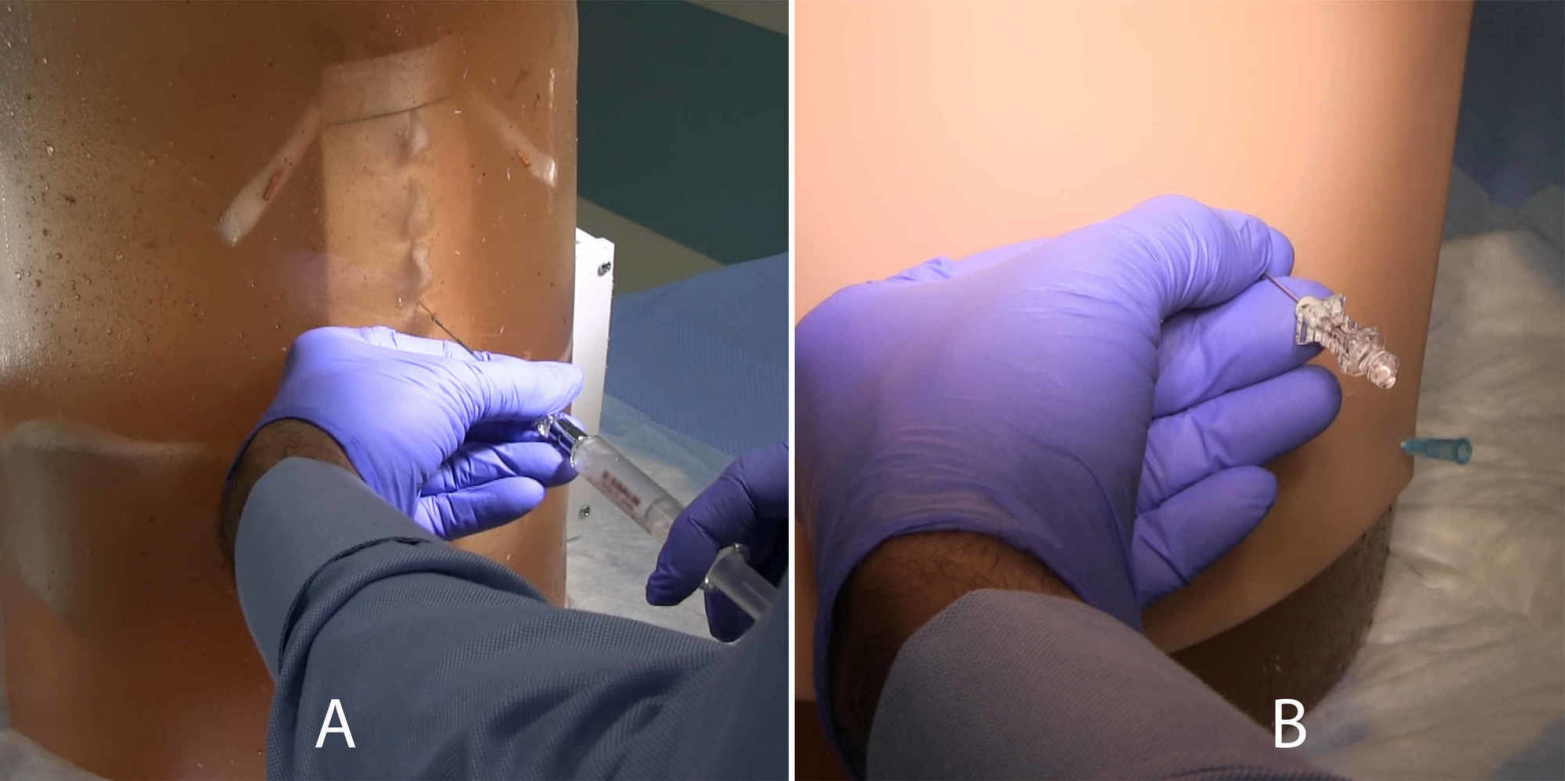
A) Operator performing simulated epidural on model. B) Operator performing simulated spinal block, note "spinal" fluid emerging from needle hub.

The completed model is shown in Figure 7. Figure 8A shows an operator using a 20g tuohy needle to perform a simulated epidural. Figure 8B shows a spinal puncture with a 22g whitacre needle (note that the silicone skin has been installed); spinal fluid can be seen emerging from the needle hub. “Spinal fluid” is created by adding saline to model via the superior spinal canal opening; the inferior opening is occluded with a rubber stopper.

## Discussion

Access to affordable 3D printing technology has resulted in increased interest in the creation of medical phantom task trainers [2]. Recent research has validated the use of these trainers in simulation education [1]. In 2013, Vaughan et al. published a review of epidural simulators. At that time, there were thirty-one implemented into clinical practice [5]. These range from very simple, non-mannequin type simulators consisting of closed-cell foam in a frame, to very elaborate simulators with haptic feed-back, virtual reality (VR) technology, fluid flow, and needle tracking [5]. Understandably, in 2013 none of the identified simulators contained user-built 3D-printed parts. The authors felt that mannequin type simulators are superior as they allow palpation of surface landmarks. We agree. Neuraxial blockade remains most frequently a “blind” approach in that surface landmarks are critical to the success of these procedures. We have designed our simulator to include palpable spinous processes as well as the iliac crests. Some of the more sophisticated (and expensive) commercially available simulators feature virtualized anatomy… the point of which is to provide feedback to the user regarding the location of the needle. One advantage of our model is that the matrix is transparent gel. Thus, the operator can easily visualize needle location and trajectory. The opaque skin allows the operator to begin the procedure using palpable landmarks and then peel back the skin to check the needle location if necessary. In morbidly obese patients ultrasound may be used to facilitate identification of bony landmarks [6]. Our current model is able to provide realistic ultrasound images. This is due to the placement of the LF. We placed the LF over the bony vertebral anatomy. Previous work by Clifton et al. has validated this construction technique for the construction of a lumbar drain simulator [7]. This has the advantage of simplifying model assembly as well as re-use. We recognize the limitation of this approach in that in vivo the LF is deep to the bony landmarks. Our model does have a realistic feel, however. Though technically challenging to construct, future iterations of this model may incorporate an LF with the spinal canal. We experimented with other materials to construct the LF; however, we felt that the EVA gave the most realistic feel when using a loss of resistance technique. And while we are currently designing a study to formally validate our model, initial impressions of tactile feel and realism were deemed positive by experienced anesthesia providers.

An additional advantage of the gel matrix is reusability. If repairs need to be made to the model, for example replacing the LF or a vertebra, one can simply cut and peel the gel from the model. The gel can then be reheated, the model placed back in the form, and the gel re-poured. As long as most of the gel has been removed, the new gel will incorporate with old.

Finally, the primary aim of our simulator was affordability (and thus availability). The finished cost of this model comes in under $500 USD. The single most expensive component is the gelatin matrix. There is an investment/startup cost to begin 3D printing. Our printer was $700 USD, un-assembled. We used a RepRap based printer by Prusa Research. Other RepRap type printers are now available for under $200, though they may be more difficult to use than the Prusa printers.

## Conclusions

3D printing technology has reached a high level of maturity and accessibility. We describe a successful technique for creating a low-cost 3D printed lumbar neuraxial block simulator. This may be useful to medical teaching and research institutions and facilities that may not have access to adequate simulation resources; the technique described herein will allow the creation of low-cost simulation models to teach lumbar neuraxial blockade. Future work will focus on continued refinement of the model features and construction.
